# Synergistic associations of antenatal care visits and iron-folic acid supplementation with low birth weight: a pooled analysis of national surveys from six south Asian countries

**DOI:** 10.1186/s12889-024-18295-2

**Published:** 2024-03-18

**Authors:** Vishnu Khanal, Sangita Bista, Shiva Raj Mishra

**Affiliations:** 1 Nepal Development Society, Bharatpur, Nepal; 2 Menzies School of Health Research, Charles Darwin University, Alice Springs, Australia; 3 Independent Public Health Consultant, Kathmandu, Nepal

**Keywords:** Low birth weight, Birth outcomes, Pooled analysis, Pregnancy, Antenatal care, Iron-folic acid

## Abstract

**Background:**

The prevalence of low birth weight (LBW) has remained high (24.9%) in the South Asian region with a significant impact on newborn survival. This region bears nearly 40% of global burden of LBW. While antenatal care (ANC) and iron-folic acid supplementation independently have been considered effective for improving maternal and newborn outcomes, the evidence on the combined effect of these two supplements on LBW is lacking. This study aimed to examine the synergistic association of ANC and iron-folic acid supplementation on LBW in the South Asian region using pooled data from six South Asian countries.

**Methods:**

Nationally representative surveys from Nepal, India, Bangladesh, Pakistan, Maldives, and Afghanistan were included in the study. Birth weight and the prevalence of LBW for singleton last-born children were reported using descriptive statistics. The association between LBW and ANC visits and the interaction between iron-folic acid consumption and ANC were examined using multiple logistic regression.

**Results:**

The mean birth weight in the region was 2841.8 g with an LBW prevalence of 17.1%. Country-specific prevalence ranged from 11.4% in Nepal to 22.4% in Pakistan. Not attending ANC visits (adjusted odds ratio (AOR): 1.24; 95% confidence interval (CI): 1.16, 1.34) and not consuming iron-folic acid (AOR: 1.14; 95% CI: 1.08, 1.21) were significantly associated with a higher likelihood of LBW. Furthermore, jointly, having < 4 ANC visits and < 180 days of iron-folic acid supplementation was associated with a higher likelihood (AOR: 1.29; 95% CI: 1.22, 1.36) of having LBW compared to those who had  ≥ 4 ANC visits and ≥ 180 days of iron-folic acid consumption after controlling for key confounding factors.

**Conclusions:**

The current study provides important evidence on the synergy between ANC visits and iron-folic acid consumption during pregnancy to capitalize on the existing national maternal health programs in the South Asian region, including low-and middle-income countries for positive foetal outcomes.

**Supplementary Information:**

The online version contains supplementary material available at 10.1186/s12889-024-18295-2.

## Background

Birth weight has long been recognized as an important predictor of newborn and infant survival. Low birth weight (LBW) is defined as a birth weight of less than 2500 g, irrespective of the gestational age [1, 2]. Previous studies have reported that LBW babies are more likely to die than their normal weight counterparts [3, 4]. Furthermore, LBW babies who do survive often fail to reach their full potential in adulthood, with a higher risk for early onset of non-communicable disease [5–7].

The global prevalence of LBW was 14.7% in 2020 [8], which varies according to the region, and low- and middle-income countries contribute to ${\sim}$ 91% of LBW cases [1, 9]. Given its sheer burden, the prevalence of LBW has been included as an indicator in the Global Nutrition Monitoring Framework and listed in the World Health Organization’s (WHO) Global reference list of 100 core health indicators [10]. Further, the World Health Assembly (WHA) has set an ambitious target to reduce LBW by 30% between 2012 and 2030, which requires an average annual reduction of 1.96% between 2012 and 2030 [8]. However, South Asia’s net reduction is 0.85% compared to 0.30% globally [8]. The region still had the highest LBW prevalence (24.9%) in 2020, despite seeing the most progress in reducing LBW prevalence and absolute numbers [8]. Of the 19.8 million LBW babies born globally in 2020, over 40% were born in South Asia alone, demonstrating the need to address the LBW issue as a high priority [8].

The cause of LBW is multifactorial. The causes and associated factors were reviewed comprehensively at a global level by Kramer in the 1980s [11]. Several studies since then have highlighted the role of improved maternal health, nutrition, socioeconomic status, education, smoking, and alcohol consumption on LBW [12, 13]. While addressing socioeconomic inequality and promoting the principles of justice within the healthcare system is a long-term solution to combat LBW [1], it is equally important to implement effective short-and mid-term strategies to bring about immediate improvements through the utilization of existing evidence-based routine services offered through most of the national health systems.

The WHO guideline on antenatal care (ANC) recommends eight ANC visits during pregnancy, with pregnant women provided with a number of services during each visit, including iron-folic acid supplementation [14]. Prior to 2016, four or more ANC visits were recommended. The antenatal period is the most suitable time to implement short-term interventions to reduce the burden of LBW. Interventions to reduce LBW are focused on improving maternal nutritional status, reducing anaemia, and reducing key morbidities such as maternal hypertension, and infections during pregnancies [15, 16]. Key interventions during pregnancy, including supplementation with folic acid, calcium, iron, vitamin D, and zinc, have been studied [15, 17]. However, the evidence has not always been conclusive. There is a paucity of evidence from South Asia on what works to reduce LBW, the chronic issue, impeding newborn survival, in the region. Iron-folic acid is one of those interventions of interest. Some studies [18, 19] have reported that iron supplementation was effective in improving birth weight. However, a recent review [15] reported that there was no significant improvement. Most of the original studies are country-specific or conducted in regions other than South Asia [12, 20]. A recent study from South Asia [21] has examined the quantity and quality of ANC in the region and its association with LBW prevalence. However, previous studies have not examined the association of iron-folic acid with LBW in South Asia. Iron-folic acid supplementation is provided during pregnancy as one of the ANC services. It is essential to examine how specific components of ANC play roles in maternal and foetal outcomes.

This study aims to investigate the synergistic association between ANC, iron-folic acid supplementation, and LBW in South Asia. By using pooled data from six countries in the region, this study seeks to provide a comprehensive understanding of the individual as well as the synergistic role of these interventions across women’s reproductive journey. Ultimately, this knowledge can inform policy decisions and guide cost-effective measures to reduce LBW in South Asia.

## Methods

### Data source

This study utilized the datasets of nationally representative Demographic and Health Surveys (DHS) from six countries in South Asia: Nepal (2022) [22], India (2019–2021) [23], Bangladesh (2017) [24], Pakistan (2017–2018) [25], Maldives (2016–2017) [26] and Afghanistan (2015) [27]. We could not include data for Sri Lanka as a recent dataset is not available publicly. For these countries, women aged 15–49 were eligible for interview with the following number of women included in the study: Nepal (*n* = 14, 845), India (*n* = 724,115), Bangladesh (*n* = 20,127), Pakistan (*n* = 12, 364), Maldives (*n* = 7, 699) and Afghanistan (*n* = 29,461). The DHS surveys are conducted every five years and use globally validated and locally adapted methodology.

The DHS follow a multi-stage cluster survey design, and sampling details can be found in the full reports of the respective surveys. The respondents are women and men from the households who reported household (family) and individual-level variables. Mothers consented and reported for themselves and their children. For this study, the ‘Children Dataset (KR)’, which contains information on birth weight and the selected variables, was used. The response rates for the surveys were more than 90% in all countries at the household level. The details of sample size and the response rate for women, men, and households can be found in publicly available DHS reports of each country.

### Variables

#### Outcome variable

This study used the global standard definition of low birth weight as less than 2500 grams [28]. In all surveys included in this analysis, birth weight was recorded using the mother’s recall and/or birth card. Birth weight was categorized into: ‘low birth weight’ if < 2500 grams and ‘non-low birth weight’ if otherwise. For this study, we limited our analysis to singleton and last-born children to facilitate interpretation and avoid the confounding effect of preterm and multiple pregnancies. The DHS data in each country has a significant proportion of last-born singleton children whose birth weights were missing, i.e. Nepal (20.0%), India (8.6%), Bangladesh (54.6%), Pakistan (80.5%), Maldives (1.8%), and Afghanistan (86.0%) (*variable: M19A in the* ‘Children Dataset (KR)’). Individuals with missing birth weights were removed from the analysis as our objective was to examine the association of iron-folic acid with low birth weight.

### Factors

The main independent variables in this study were antenatal care and number of days of iron-folic acid consumption, both of which are key services provided during pregnancy across the South Asian region. The number of ANC visits was reported as a continuous variable and categorized into binary: ‘≥ 4 visits’, and ‘<4 ANC’ based on widely accepted recommendations of ‘4 or more ANC visits’ prior to 2016 [29]. The number of days mothers consumed iron-folic acid during pregnancy was a continuous variable and categorized into: ‘≥ 180 days’ and ‘<180 days’. To ascertain the synergistic association with LBW, these two binary variables were combined into a new variable as below:

- ≥ 4 visits and ≥ 180 days of iron-folic acid (*most preferred*).
- ≥ 4 visits and < 180 days of iron-folic acid;
- < 4 visits and ≥ 180 days of iron-folic acid;
- < 4 visits and < 180 days of iron-folic acid *(least preferred*).

We also used ANC and iron-folic acid supplementation as separate variables, as mostly cited in the existing literature. For ANC visits, we categorized into three groups: 4 or more ANC, 1–3 ANC, and No ANC. Similarly, we categorized iron-folic acid supplementation into three categories: more than 90 days, 1–90 days, and none [30].

Several confounding factors were considered, including family wealth (poorest, poor, middle, richer, and richest); the place of residence (urban or rural); maternal age (15–19 years; 20–34 years; ≥ 35 years), maternal and paternal education (no formal education; primary; secondary; higher), maternal smoking (yes; no) and birth order (primipara; multipara). In addition, the type of cooking fuel was categorized as polluting (kerosene, coal, ignite, charcoal, wood, straw, agricultural crop, animal dung) and non-polluting (biogas, electricity, natural gases, LPG). Family wealth is a composite indicator based on the family possession of different asset variables [31]. A score is assigned for possessing each asset, and a principal component analysis is used to calculate the wealth score, and finally dividing into five quintiles: poorest, poor, middle, rich, and richest. The details of such asset possessions are provided in respective DHS reports [22–27].

### Statistical analysis

Descriptive statistics were used to report the mean birthweight (in grams) along with their 95% confidence interval (CI) as well as the prevalence of LBW and its frequency distribution. The association between independent variables and LBW was determined first by using a Chi-square test (χ^2^). Subsequently, the significant variables (*p* < 0.05) were included in multiple logistic regression models. Adjusted odds ratios (AOR), together with their corresponding 95% CI, were reported.

For the two key variables of interest, we first examined the association of ANC and iron-folic acid in multiple logistic regression (Table 3, Model a). We further examined the association of binary variables for ANC (≥ 4 visits, and < 4 ANC) and iron-folic acid consumption (≥ 180 days and < 180 days) in multiple logistic regression (Table 3, Model b). Finally, we examined the synergistic relationship of ANC and iron-folic acid in multiple logistic regression (Table 3, Model c). In all analyses, the confounding variables were selected based on Chi-square test (χ^2^) (Full results presented in the Supplementary Tables).

We also performed sensitivity analysis as the dataset had a large number of missing birthweights. Firstly, we investigated if key independent variables were associated with the reporting of the birth weight (yes/no). Secondly, we conducted multiple additional analysis to check if the association of ANC visit, iron-folic acid consumption and their combined variables remained significant after controlling for other factors, including the source of birthweight. A p-value of < 0.05 was set as statistically significant. All statistical analyses were performed using Statistical Package for Social Science Version 28 (IBM Corp, Armonk, NY, USA) after accounting for the sampling weight using complex sample analysis.

### Ethics

This study used publicly available de-identified datasets made available through The DHS Program (https://dhsprogram.com/data/available-datasets.cfm). The DHS surveys were approved by the respective ethics committees in the individual countries and by ICF Macro Institutional Review Board in Calverton, Maryland, USA. A further analysis protocol was also approved by the DHS Program prior to allowing access to the data. The individual reports from the respective countries outline the consent process that adheres to the ethical standards.

## Results

### Birth weight and prevalence of low birth weight

**Fig. 1:**
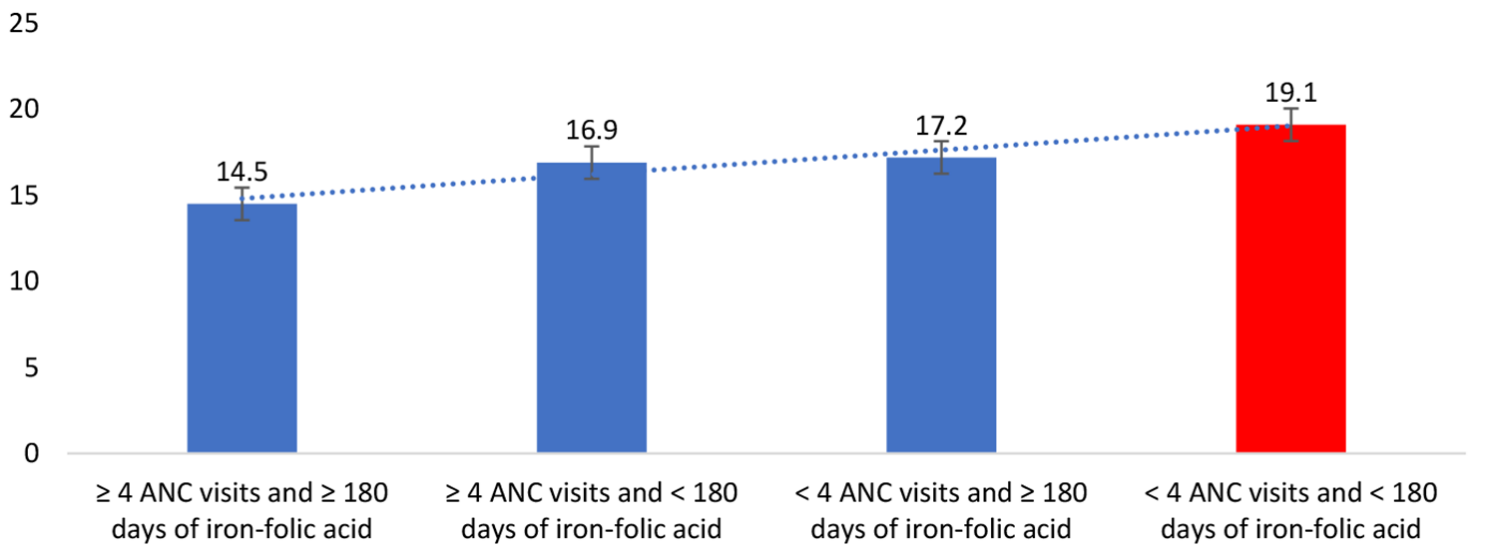
Prevalence of low birth weight by joint ANC visit and iron-folic acid consumption in the South Asian region

Table 1 presents the mean birth weight in the South Asia region. The mean birth weight was 2841 g (95% CI: 2839.0, 2844.5 g), with a prevalence of LBW of 17.1%. The prevalence of LBW ranged between 11.4 and 22.4% across the region. The prevalence of LBW was 19.1% among the mothers who had reported < 4 ANC visits and < 180 iron-folic acid consumption compared to 14.5% among those who reported ≥ 4 visits and ≥ 180 days (Fig. 1).

**Table 1:** Average birth weight and burden of low birth weight in South Asian region (*N* = 171,213)

	South Asian region	Nepal	India	Bangladesh	Pakistan	Maldives	Afghanistan
Average birth weight (95% confidence interval) in grams	2841.8 (2839.0; 2844.5)	3001.5 (2977.8; 3025.7)	2827.1 (2824.3; 2829.9)	3012.9 (2984.7; 3041.2)	2970.0 (2929.3; 3010.7)	3030.4 (3009.1; 3051.7)	3167.4 (3137.2; 3197.5)
Number of birth weights reported*	171,213	2238	160,109	2249	1185	2593	2739
Low birth weight ** n (%)	27,915 (17.1)	272 (11.4)	26,366 (17.0)	319 (14.6)	224 (22.4)	329 (11.9)	405 (15.3)

*Singleton and lastborn. **weighted prevalence rate

### Characteristics of participants

Table 2 presents the characteristics of participants by key variables included in the study. There were slightly high percentage male births (53.4%) compared to females. The majority of the mothers were within the age group of 20–34 years (87.3%), and the most of them resided in rural areas (69.8%). A total of 51.4% reported using polluting cooking fuel. One-third of births were primiparous, and 60.5% reported attending the recommended four or more ANC visits during pregnancy. A small proportion of mothers reported not consuming any iron-folic acid (14.5%), while 46.2% reported consuming it for more than 90 days. Furthermore, only 27.7% of mothers reported consuming iron-folic acid supplementation for 180 days or more during pregnancy as per the recommendation. The intercountry iron-folic acid consumption for 180 days or more varied significantly (χ^2^ p-value < 0.001) within the region: Nepal (69.2%), India (27.5%), Bangladesh (23.0%), Pakistan (22.6%), Maldives (36.3%), Afghanistan (3.3%).

Regarding joint ANC and iron-folic acid consumption, only 20.9% reported attending 4 or more ANC visits and consuming iron-folic acid for 180 days or more. Almost one-third (32.7%) reported attending < 4 ANC visits and consuming iron-folic acid for < 180 days (Table 1).

**Table 2:** Distribution of low birth weight by participant characteristics in the South Asian region (*N* = 171,213)

Variables	Total N (%)	Low birth weight (< 2500 g) N (%)
**Sex of child**		*p* < 0.001
Male	91,986 (53.9)	13,977 (15.8)
Female	79,227 (46.1)	13,938 (18.6)
**Maternal age**		*p* < 0.001
15–19 years	5248 (3.4)	1093 (21.7)
20–34 years	147,784 (87.3)	24,152 (17.1)
35 years and above	18,181 (9.3)	2670 (15.9)
**Maternal education**		*p* < 0.001
No formal education	31,745 (17.6)	5976 (19.4)
Primary	21,052 (11.9)	3817 (19.7)
Secondary	90,965 (52.4)	14,621 (17.1)
Higher	27,451 (18.2)	3501 (13.1)
**Paternal education**		*p* < 0.001
No formal education	140,788 (82.3)	23,337 (17.3)
Primary	5619 (3.2)	912 (17.5)
Secondary	18,318 (10.5)	2810 (16.4)
Higher	6488 (4.0)	856 (13.9)
**Household wealth**		*p* < 0.001
Poorest	38,916 (20.2)	7445 (20.3)
Poor	38,395 (20.5)	6513 (18.6)
Middle	34,629 (20.0)	5359 (16.6)
Richer	31,787 (20.2)	4752 (15.7)
Richest	27,486 (19.1)	3846 (14.0)
**Place of residence**		*p* < 0.001
Urban	39,981 (30.2)	6166 (16.1)
Rural	131,232 (69.8)	21,749 (17.5)
**Cooking fuel**		*p* < 0.001
Non-polluting	80,490 (51.4)	11,943 (15.7)
Polluting	90,719 (48.6)	15,971 (18.6)
**Maternal smoking**		*p* < 0.001
Yes	9631 (3.1)	1466 (20.2)
No	159,330 (96.9)	26,130 (17.0)
**Birth order**		*p* < 0.001
Primipara	60,572 (35.9)	10,623 (18.3)
Multipara	110,641 (64.1)	17,292 (16.4)
**ANC visits**		*p* < 0.001
≥ 4 visits	102,666 (60.5)	15,691 (16.0)
1–3 visits	56,487 (32.2)	9917 (18.4)
No visit	12,060 (7.3)	2307 (20.4)
**Iron-folic acid consumptions in days**		*p* < 0.001
> 90 days	76,892 (46.2)	11,526 (15.7)
1–90 days	68,894 (39.3)	11,764 (18.1)
None	25,427 (14.5)	4625 (18.8)
**Variables for additional analysis**		
**Number of antenatal visits**		*p* < 0.001
≥ 4 visits	102,666 (60.5)	15,691 (16.0)
< 4 visits	68,547 (39.5)	12,224 (18.8)
**Iron-folic acid consumption in days**		*p* < 0.001
≥ 180 days	44,342 (27.7)	6555 (15.1)
< 180 days	126,871 (72.3)	21,360 (17.9)
**Antenatal visits and iron-folic acid consumption**		*p* < 0.001
≥ 4 visits and ≥ 180 days	33,145 (20.9)	4715 (14.5)
≥ 4 visits and < 180 days	69,521 (39.5)	10,976 (16.9)
< 4 visits and ≥ 180 days	11,197 (6.7)	1840 (17.2)
< 4 visits and < 180 days	57,350 (32.7)	10,384 (19.1)

P: Chi-square p-value. ANC: Antenatal care

### Association of antenatal care and Iron-folic acid consumption with low birth weight

Table 3 presents the individual associations of ANC and iron-folic acid consumption with LBW. Mothers who reported having no ANC (AOR: 1.24; 95% CI: 1.16, 1.34), and 1–3 ANC visits (AOR: 1.09; 95% CI: 1.05, 1.14) had a higher likelihood of LBW compared to those who reported having 4 or more ANC visits. Similarly, mothers who reported not consuming iron-folic acid (AOR: 1.14; 95% CI: 1.08, 1.21) and consuming for 1–90 days (AOR: 1.11; 95% CI: 1.06, 1.16) were also found to have a higher likelihood of LBW compared to those who reported consuming for more than 90 days.

Model b (Table 3) illustrates the independent association of ANC visits and iron-folic acid consumption. Mothers who reported having less than 4 ANC visits (AOR: 1.13; 95% CI: 1.08, 1.18) and less than 180 days of iron-folic acid consumption (AOR: 1.13; 95% CI: 1.08, 1.18) had a higher likelihood of having an LBW child.

The combined variable of ANC visits and iron-folic acid consumption also showed similar results (Model c). Mothers who reported having < 4 ANC visits and < 180 days of iron-folic acid (AOR: 1.29; 95% CI: 1.22, 1.36), < 4 ANC visits and ≥ 180 days of iron-folic acid (AOR: 1.18; 95% CI: 1.08, 1.3), ≥ 4 visits and < 180 days of iron-folic acid (AOR: 1.15; 95% CI: 1.09, 1.22) had a higher likelihood of having a LBW compared to those with ≥ 4 visits and ≥ 180 days of iron-folic acid (most preferred) (Table 3 Model c). Full results of the models are presented in Supplementary Tables (1, 2 and 3).

**Table 3:** Association of ANC visits and iron-folic acid consumption with low birth weight in the South Asian region

Model	Adjusted odds ratio (95% confidence interval)*
**Model a**	
**ANC visits**	*p* < 0.001
≥ 4 visits	1.0
1–3 visits	1.09 (1.05, 1.14)
No visit	1.24 (1.16, 1.34)
**Iron-folic acid consumption**	*p* < 0.001
> 90 days	1.00
1–90 days	1.11 (1.06, 1.16)
None	1.14 (1.08, 1.21)
**Model b**	
**Number of antenatal visits**	*p* < 0.001
4 or more ANC	1.00
Less than 4 ANC	1.13 (1.08, 1.18)
**Iron-folic acid consumption**	*p* < 0.001
≥ 180 days	1.00
< 180 days	1.13 (1.08, 1.18)
**Model c**	
**ANC visits and iron-folic acid consumption**	*p* < 0.001
≥ 4 visits and ≥ 180 days	1.0
≥ 4 visits and < 180 days	1.15 (1.09, 1.22)
< 4 visits and ≥ 180 days	1.18 (1.08, 1.3)
< 4 visits and < 180 days	1.29 (1.22, 1.36)

*Model a to c were adjusted for infant sex, maternal age, maternal education, paternal education, household wealth, place of residence, type of cooking fuel, maternal smoking, and birth order. Full results shown in supplementary tables. ANC: Antenatal care

We performed sensitivity analysis to examine if the reported association was affected by the missing birth weight reports. We identified that there was significant difference in the birth weight recording by key sample characteristics (Supplementary Table 4). The proportionate distribution of missing birthweight data was not similar across all sub-sections of mothers. For instance, the missing birth weight was 79.4% in rural areas compared to just 20.4% in urban areas. Similarly, those with higher education and those from the richest families had the least amount of missing data.

We conducted additional analysis using the source of birth weight data (*variable M19A*). We re-ran the analysis of model mentioned above and added the source of birth weight. Mothers who reported having < 4 ANC visits and < 180 days of iron-folic acid (AOR: 1.27; 1.20, 1.35); < 4 ANC visits and ≥ 180 days of iron-folic acid (AOR: 1.19; 1.08, 1.30), ≥ 4 ANC visits and < 180 days of iron-folic acid (AOR: 1.15; 1.09, 1.22) had a higher likelihood of having a LBW child compared to those who reported ≥ 4 visits and ≥ 180 days of iron-folic acid (most preferred).

We ran similar analysis of model a, keeping ANC and iron-folic acid as individual variables. Upon adding the source of birth weight data variable (*variable M19A*), there was no change in the association of ANC visit and iron-folic acid consumption. Mothers who reported having no ANC (AOR:1.24; 95% CI: 1.15, 1.34), and 1–3 ANC visits (AOR:1.09; 95% CI: 1.04, 1.14) had a higher likelihood of having a LBW child compared to those who reported having 4 or more ANC visits. Similarly, mothers who reported not consuming iron-folic acid (AOR: 1.13; 95% CI:1.07, 1.20) and consuming for 1–90 days (AOR: 1.10; 95% CI: 1.06, 1.15) were also found to have a higher likelihood of having an LBW child compared to those who reported consuming it for more than 90 days.

Additionally, we re-categorized the birth weight reported (Yes/No) as source of birth weight variables. While running the similar analysis as mentioned above, we found no change in the association. ANC visits, iron-folic acid consumption and their combined variable still remained significantly associated with LBW after controlling for other factors in the models (model a, and model c).

## Discussion

This study utilized data from the NDHS in six South Asian countries to investigate the relationship between ANC visits and iron-folic acid consumption and their combined effect on LBW prevalence. The study found that adhering to the recommended ANC visits and consuming iron-folic acid for the recommended duration can be protective against LBW. The protective effect was even stronger when minimum adherence to both is met. The fact that the interaction term of ANC and iron-folic acid was not significant, but ANC and iron-folic supplementation was significant in additional analysis, shows that the effect of ANC and iron-folic are separate from each other, and their combined effect is only additive (no interaction). These findings are consistent with previous studies conducted in South Asia [13] and other low and middle-income countries [12]. A multi-country study from Sub-Saharan Africa also reported a lower likelihood of LBW birth among those who had one or more ANC visits compared to those with no visits [32]. Studies from Nepal, India, Pakistan, and Bangladesh have reported that mothers who attended less than the recommended ANC visits had a higher likelihood of giving birth to LBW babies [19, 33–35].

The causal mechanism of how ANC helps improve birth weight is not clearly understood. However, there is plausible explanation. Health services offered during ANC visits include tracing maternal medical history, measuring blood pressure, checking for anemia, and examining fetal movement, as well as screening, tests, treatment of syphilis and bacteriuria if indicated, preventive measures such as tetanus toxoid immunization and iron-folic acid supplementation, and general health education, can drive better foetal outcomes [3]. The ANC visits are also used as an opportunity to educate pregnant women and their partners or accompanying family members on different areas such as nutrition, birth preparedness, recognizing danger signs during pregnancy, and importance of subsequent ANC visits. In collective societies such as South Asia, education and empowerment of the significant others are important for improving general health, well-being, and pregnancy outcomes, and enhancing foetal growth.

Iron-folic acid supplementation also appeared to have a consistent association with LBW, both independently as well as synergistically with ANC visits. Previous studies have reported on the association between iron-folic acid supplementation and the risk of LBW [13, 19, 36, 37], although another review suggested that there was no association [38]. More recently, a systematic review suggested that consumption of iron-folic acid during pregnancy was associated with a higher birth weight (mean difference: 0.18 kg; 95% CI: 0.00, 0.36 kg), as well as a lower likelihood of LBW (relative risk: 0.85; 95% CI: 0.58, 1.38), although the latter association was not statistically significant [15].

Iron-folic acid supplementation during pregnancy can help reduce oxidative stress on the foetus and improve hormonal and neuronal regulation of pregnancy and oxygenation to the foetus [17]. Iron deficiency anaemia can cause changes in the level of stress hormones such as norepinephrine, cortisol, and corticotropin, resulting in oxidative stress and foetal growth restriction. Iron supplementation helps reverse such stress [17, 39]. For instance, a previous study has reported that six weeks of iron supplementation had shown a significant reduction in oxidative stress biomarkers such as malondialdehyde, glutathione peroxidase (GSH-Px), superoxide dismutase (SOD) and catalase (*p* < 0.005) [40]. Poor appetite during pregnancy has been reported among 49.5% of pregnant women and even higher (60.6%) in the first trimester of pregnancy in a large observational cohort study from Nepal (*n* = 3623) [41]. Consequently, the mean birth weight was lower among mothers who had experienced nausea, vomiting, and poor appetite during pregnancy compared to those with no symptoms (2768 g vs. 2807 g; *p* = 0.27). Iron-folic acid supplementation has been found useful in improving mother’s appetite leadings to better intake of food, consequently to improved foetal outcomes. For instance, a review from low and middle income countries summarized that the intake of local food of 1500 kcal per day was associated with a reduced likelihood of LBW (RR: 0.17; 95% CI: 0.09, 0.29) [17].

The major strength of this study is its use of pooled data from the six countries of South Asia with very similar cultural and regional contexts and the use of pooled data based on nationally representative samples using locally validated questionnaires. The individual surveys have high response rates. Robust analysis was conducted, including sensitivity analysis, to ensure correct reporting of association. However, several limitations should be acknowledged. First, missing values were present for the birth weight in each country which may have an impact on our findings as some countries have more missing values than others. Second, the birth weight was recorded primarily by mothers’ recall or child cards when available. While health facility record is an option, in reality, the health facility data are often poorly recorded and managed, and a very small portion, mainly for the tertiary hospitals, are digitalized; therefore, it is not possible to collect data from the health facility’s record. Furthermore, the mother’s recall of size at birth is an alternative that has been previously used [42, 43]. However, the perceived size at birth and birth weight agreement is also questionable, with only 67.2% agreement, according to a recent NDHS analysis from Nepal [44]. Therefore, maternal recall of birth weight is the most feasible in the South Asian context. Finally, the Indian population in this study has the largest population as shown in Table 2. This may have influenced the result to some degree. However, our aim was to examine the association at the South Asian region level, not at the country level. Future studies may need to examine if the same hypothesis holds true within the countries. South Asian countries are unique, and they have intra-country variations by culture, geography, and access to nutrition and health care. Additionally, many confounding factors were not included in the study due to the generic nature of DHS surveys which are not specially focused on LBW. The DHS studies use a cross-sectional study design. Therefore, our study can only ascertain association, not causation. Notwithstanding these limitations, the findings suggest an opportunity to reduce the burden of LBW by reinforcing the use of ANC visits and iron-folic acid supplementation within existing health service delivery.

## Conclusions

In conclusion, both ANC visits and iron-folic acid supplementation were associated with a reduced prevalence of LBW, and their combined association was even more significant when minimum adherence for both was utilized together as per recommendation. These findings suggest the need to improve access to ANC and tailor iron-folic acid supplementation to adhere to the recommendation.

### Electronic supplementary material

Below is the link to the electronic supplementary material.

Supplementary Material 1

## Data Availability

This study uses the publicly available de-identified data which can be obtained from The DHS Program (https://dhsprogram.com/data/available-datasets.cfm).

## References

[1] WHO. Global nutrition targets 2025: low birth weight policy brief. World Health Organization; 2014.

[2] Global nutrition targets. 2025. Low birth weight policy brief. Geneva; 2023.

[3] Katz J, Lee AC, Kozuki N, Lawn JE, Cousens S, Blencowe H (2013). Mortality risk in preterm and small-for-gestational-age infants in low-income and middle-income countries: a pooled country analysis. Lancet.

[4] Kc A, Wrammert J, Nelin V, Ewald U, Clark R, Målqvist M (2015). Level of mortality risk for babies born preterm or with a small weight for gestation in a tertiary hospital of Nepal. BMC Public Health.

[5] WHO. Born too soon: the global action report on preterm birth. 2012.

[6] Gu H, Wang L, Liu L, Luo X, Wang J, Hou F (2017). A gradient relationship between low birth weight and IQ: a meta-analysis. Sci Rep.

[7] Belbasis L, Savvidou MD, Kanu C, Evangelou E, Tzoulaki I (2016). Birth weight in relation to health and disease in later life: an umbrella review of systematic reviews and meta-analyses. BMC Med.

[8] UNICEF. Low birthweight New York: UNICEF. 2023 [Available from: https://data.unicef.org/topic/nutrition/low-birthweight/#1.

[9] Blencowe H, Krasevec J, De Onis M, Black RE, An X, Stevens GA (2019). National, regional, and worldwide estimates of low birthweight in 2015, with trends from 2000: a systematic analysis. Lancet Global Health.

[10] WHO. Low birth weight Geneva: World Health Organization. 2023 [Available from: https://www.who.int/data/nutrition/nlis/info/low-birth-weight.

[11] Kramer MS (1987). Determinants of low birth weight: methodological assessment and meta-analysis. Bull World Health Organ.

[12] Arsyi M, Besral B, Herdayati M, Phalkey R (2022). Antenatal Care Services and incidence of low Birth Weight: a comparison of demographic and health surveys in 4 ASEAN Countries. J Prev Med Public Health.

[13] Sathi NJ, Ahammed B, Alam K, Hashmi R, Lee KY, Keramat SA (2022). Socioeconomic inequalities in low birth weight in South Asia: a comparative analysis using demographic and health surveys. SSM-Population Health.

[14] Joshi C, Torvaldsen S, Hodgson R, Hayen A (2014). Factors associated with the use and quality of antenatal care in Nepal: a population-based study using the demographic and health survey data. BMC Pregnancy Childbirth.

[15] Katharina da Silva L, Erika O, Prakash S, Amarjargal D, Olukunmi Omobolanle B, Juan Pablo P-R (2017). Effects of nutrition interventions during pregnancy on low birth weight: an overview of systematic reviews. BMJ Global Health.

[16] Piso B, Zechmeister-Koss I, Winkler R (2014). Antenatal interventions to reduce preterm birth: an overview of Cochrane systematic reviews. BMC Res Notes.

[17] Park J, Harari O, Siden E, Zoratti M, Dron L, Zannat N et al. Interventions to improve birth outcomes of pregnant women living in low- and middle-income countries: a systematic review and network meta-analysis [version 2; peer review: 1 approved, 2 approved with reservations]. Gates Open Res. 2020;3(1657). 10.12688/gatesopenres.13081.1 PMC7520556 33134854

[18] Balarajan Y, Subramanian S, Fawzi WW (2013). Maternal iron and folic acid supplementation is associated with lower risk of low birth weight in India. J Nutr.

[19] Khanal V, Zhao Y, Sauer K (2014). Role of antenatal care and iron supplementation during pregnancy in preventing low birth weight in Nepal: comparison of national surveys 2006 and 2011. Archives Public Health.

[20] Weyori AE, Seidu A-A, Aboagye RG, Holmes FA, Okyere J, Ahinkorah BO (2022). Antenatal care attendance and low birth weight of institutional births in sub-saharan Africa. BMC Pregnancy Childbirth.

[21] Neupane S, Scott S, Piwoz E, Kim SS, Menon P, Nguyen PH (2023). More is not enough: high quantity and high quality antenatal care are both needed to prevent low birthweight in South Asia. PLOS Global Public Health.

[22] New ERA, Ministry of Health and Population [Nepal] (2023). Nepal Demographic and Health Survey 2022.

[23] IIPS ICF (2022). National Family Health Survey (NFHS-5), 2019-21: India: Volumn I.

[24] NIPORT ICF. Bangladesh Demographic and Health Survey 2017-18. Dhaka, Bangladesh and Rockville, Maryland, USA: National Institute of Population Research and Training (NIPORT) ICF; 2020.

[25] NIPS ICF (2019). Pakistan Demographic and Health Survey 2017–2018. Islamabad, Pakistan and Rockville.

[26] Ministry of Health (MOH). [Maldives], ICF. Maldives Demographic and Health Survey 2016-17. Male, Maldives and Rockville, Maryland, USA; 2018.

[27] Central Statistics Organization (CSO) (2016). Ministry of Public Health (MoPH), ICF. Afghanistan Demographic and Health Survey 2015.

[28] WHO. Preterm and low birth weight infants 2023 [Available from: Newborn Health (who.int).

[29] Khanal V, Bista S, Mishra SR, Lee AH (2023). Dissecting antenatal care inequalities in western Nepal: insights from a community-based cohort study. BMC Pregnancy Childbirth.

[30] Sharma SR, Giri S, Timalsina U, Bhandari SS, Basyal B, Wagle K (2015). Low birth weight at term and its determinants in a tertiary hospital of Nepal: a case-control study. PLoS ONE.

[31] Rutstein S, Staveteig S. Making the Demographic and Health Surveys Wealth Index Comparable. Maryland, USA ICF International; 2014.

[32] Tessema ZT, Tamirat KS, Teshale AB, Tesema GA (2021). Prevalence of low birth weight and its associated factor at birth in Sub-saharan Africa: a generalized linear mixed model. PLoS ONE.

[33] Mumbare SS, Maindarkar G, Darade R, Yenge S, Tolani MK, Patole K (2012). Maternal risk factors associated with term low birth weight neonates: a matched-pair case control study. Indian Pediatr.

[34] Pawar A, Kumar D (2017). Maternal factors associated with low birth weight: a case control study in rural Kerala. Int J Community Med Public Health.

[35] Khan JR, Islam MM, Awan N, Muurlink O (2018). Analysis of low birth weight and its co-variants in Bangladesh based on a sub-sample from nationally representative survey. BMC Pediatr.

[36] WHO. WHO recommendations on antenatal care for a positive pregnancy experience. World Health Organization; 2016. 28079998

[37] Peña-Rosas JP, De‐Regil LM, Garcia‐Casal MN, Dowswell T. Daily oral iron supplementation during pregnancy. Cochrane Database Syst Reviews. 2015(7). 10.1002/14651858.CD004736.pub5 PMC8918165 26198451

[38] Imdad A, Bhutta ZA (2012). Routine Iron/Folate supplementation during pregnancy: effect on maternal anaemia and birth outcomes. Paediatr Perinat Epidemiol.

[39] Khoshfetrat MR, Mohammadi F, Mortazavi S, Rashidi A, Neyestani T, Kalantari N (2013). The effect of iron–vitamin C co-supplementation on biomarkers of oxidative stress in iron-deficient female youth. Biol Trace Elem Res.

[40] Kurtoglu E, Ugur A, Baltaci AK, Undar L (2003). Effect of iron supplementation on oxidative stress and antioxidant status in iron-deficiency anemia. Biol Trace Elem Res.

[41] Regodón Wallin A, Tielsch JM, Khatry SK, Mullany LC, Englund JA, Chu H (2020). Nausea, vomiting and poor appetite during pregnancy and adverse birth outcomes in rural Nepal: an observational cohort study. BMC Pregnancy Childbirth.

[42] Khanal V, Sauer K, Karkee R, Zhao Y (2014). Factors associated with small size at birth in Nepal: further analysis of Nepal demographic and Health Survey 2011. BMC Pregnancy Childbirth.

[43] Sreeramareddy CT, Shidhaye RR, Sathiakumar N (2011). Association between biomass fuel use and maternal report of child size at birth-an analysis of 2005-06 India Demographic Health Survey data. BMC Public Health.

[44] Acharya P, Adhikari S, Adhikari TB (2023). Mother’s perception of size at birth is a weak predictor of low birth weight: evidence from Nepal demographic and Health Survey. PLoS ONE.

