# Supplementary material for: Synergistic associations of antenatal care visits and iron-folic acid supplementation with low birth weight: a pooled analysis of national surveys from six south Asian countries
Source: BMC Public Health. 2024 Mar 18;24:835. doi: 10.1186/s12889-024-18295-2 (PMC10946146; doi:10.1186/s12889-024-18295-2)
Supplement: Supplementary file 1 — Supplementary Material 1 [file 12889_2024_18295_MOESM1_ESM.docx]

**Supplementary Table 1 (model a). Influence of ANC and iron-folic acid consumption on the likelihood of low birth weight**

| **Variables** | **Adjusted odds ratio**  **(95% confidence interval)** |
| --- | --- |
| **ANC visits** | p<0.001 |
| ≥ 4 visits | 1.00 |
| 1-3 visits | 1.09 (1.05, 1.14) |
| No visit | 1.24 (1.16, 1.34) |
| **Iron-folic acid consumption** | p<0.001 |
| > 90 days | 1.00 |
| 1- 90 days | 1.11 (1.06, 1.16) |
| None | 1.14 (1.08, 1.21) |
| **Sex of child** | p<0.001 |
| Male | 1.0 |
| Female | 1.22 (1.18, 1.27) |
| **Maternal age** | p=0.001 |
| 20-34 years | 1.0 |
| 15-19 years | 1.16 (1.05, 1.28) |
| 35 years and above | 0.93 (0.87, 0.99) |
| **Maternal education** | p<0.001 |
| Higher | 1.0 |
| No formal education | 1.41 (1.30, 1.53) |
| Primary | 1.49 (1.38, 1.61) |
| Secondary | 1.29 (1.22, 1.37) |
| **Paternal education** | p=0.165 |
| Higher | 1.0 |
| No formal education | 1.06 (0.94, 1.18) |
| Primary | 0.98 (0.83, 1.14) |
| Secondary | 0.99 (0.88, 1.13) |
| **Household wealth** | p<0.001 |
| Richest | 1.0 |
| Poorest | 1.33 (1.21, 1.46) |
| Poor | 1.25 (1.15, 1.36) |
| Middle | 1.12 (1.04, 1.21) |
| Richer | 1.08 (1.00, 1.17) |
| **Place of residence** | p<0.001 |
| Urban | 1.0 |
| Rural | 0.91 (0.86, 0.95) |
| **Cooking fuel** | p=0.196 |
| Non-polluting | 1.00 |
| Polluting | 1.03 (0.98, 1.08) |
| **Maternal smoking** | p=0.35 |
| No | 1.00 |
| Yes | 1.10 (1.01, 1.20) |
| **Birth order** | p<0.001 |
| Multipara | 1.00 |
| Primipara | 1.23 (1.18, 1.28) |

**Supplementary Table 2 (model b). Influence of ANC and iron-folic acid consumption on the likelihood of low birth weight**

| **Variables** | **Adjusted odds ratio**  **(95% confidence interval)** |
| --- | --- |
| **Number of antenatal visits** | p<0.001 |
| ≥ 4 visits | 1.00 |
| < 4 visits | 1.13 (1.08, 1.18) |
| **Iron-folic acid consumption** | p<0.001 |
| ≥ 180 days | 1.00 |
| <180 days | 1.13 (1.08, 1.18) |
| **Sex of child** | p<0.001 |
| Male | 1.0 |
| Female | 1.12 (1.18, 1.27) |
| **Maternal age** | p=0.001 |
| 20-34 years | 1.0 |
| 15-19 years | 1.16 (1.05, 1.28) |
| 35 years and above | 0.93 (0.87, 0.99) |
| **Maternal education** | p<0.001 |
| Higher | 1.0 |
| No formal education | 1.42 (1.31, 1.53) |
| Primary | 1.49 (1.38, 1.61) |
| Secondary | 1.29 (1.22, 1.37) |
| **Paternal education** | p=0.206 |
| Higher | 1.0 |
| No formal education | 1.05 (0.94, 1.18) |
| Primary | 0.98 (0.84, 1.15) |
| Secondary | 0.99 (0.88, 1.13) |
| **Household Wealth** | p<0.001 |
| Richest | 1.0 |
| Poorest | 1.33 (1.21, 1.46) |
| Poor | 1.24 (1.14, 1.35) |
| Middle | 1.12 (1.04, 1.21) |
| Richer | 1.08 (1.00, 1.17) |
| **Place of residence** | p<0.001 |
| Urban | 1.0 |
| Rural | 0.90 (0.86, 0.95) |
| **Cooking fuel** | p=0.226 |
| Non-polluting | 1.00 |
| Polluting | 1.03 (0.98, 1.08) |
| **Maternal smoking** | p=0.039 |
| No | 1.00 |
| Yes | 1.09 (1.00, 1.20) |
| **Birth order** | p<0.001 |
| Multipara | 1.00 |
| Primipara | 1.23 (1.18, 1.28) |

**Supplementary Table 3 (model c). Synergistic influence of ANC and iron-folic acid consumption on the likelihood of low birth weight**

| **Variables** | **Adjusted odds ratio**  **(95% confidence interval)** |
| --- | --- |
| **ANC visits and iron-folic acid consumption** | p<0.001 |
| ≥ 4 visits and ≥ 180 days | 1.0 |
| ≥ 4 visits and < 180 days | 1.15 (1.09, 1.22) |
| < 4 visits and ≥ 180 days | 1.18, 1.08, 1.3) |
| < 4 visits and < 180 days | 1.29 (1.22, 1.36) |
| **Sex of child** | p<0.001 |
| Male | 1.0 |
| Female | 1.22 (1.18, 1.27) |
| **Maternal age** | p=0.001 |
| 20-34 years | 1.0 |
| 15-19 years | 1.16 (1.05. 1.28) |
| 35 years and above | 0.93 (0.87,0.99) |
| **Maternal education** | p<0.001 |
| Higher | 1.0 |
| No formal education | 1.42 (1.31,1.54) |
| Primary | 1.49 (1.38, 1.61) |
| Secondary | 1.29 (1.22, 1.37) |
| **Paternal education** | p=0.212 |
| Higher | 1.0 |
| No formal education | 1.05 (0.94, 1.18) |
| Primary | 0.98 (0.84, 1.15) |
| Secondary | 0.99 (0.88, 1.13) |
| **Household wealth** | p<0.001 |
| Richest | 1.0 |
| Poorest | 1.33 (1.21, 1.56) |
| Poor | 1.24 (1.14, 1.35) |
| Middle | 1.12 (1.34, 1.21) |
| Richer | 1.08 (1.00, 1.17) |
| **Place of residence** | p<0.001 |
| Urban | 1.0 |
| Rural | 0.90 (0.86, 0.95) |
| **Cooking fuel** | p=0.223 |
| Non-polluting | 1.00 |
| Polluting | 1.03 (0.98, 1.08) |
| **Maternal smoking** | p=0.040 |
| No | 1.00 |
| Yes | 1.10 (1.00, 1.20) |
| **Birth order** | p<0.001 |
| Multipara | 1.00 |
| Primipara | 1.23 (1.18, 1.28) |

**Supplementary Table 4. Comparison of characteristics of samples with and without birthweight data**

| **Key variables** | **Birth weight reported** | | **P-value** |
| --- | --- | --- | --- |
|  | **No (%)** | **Yes (%)** |  |
| **Place of residence** |  |  | <0.001 |
| Urban | 8813 (20.4) | 39983 (30.2) |  |
| Rural | 31972 (79.6) | 131232 (69.8) |  |
| **Household wealth** |  |  | <0.001 |
| Poorest | 13544 (32.2) | 38917 (20.2) |  |
| Poor | 9928 (22.8) | 38395 (20.5) |  |
| Middle | 7422 (18.7) | 34629 (20.0) |  |
| Richer | 6186 (15.8) | 31787(20.2) |  |
| Richest | 3705 (10.5) | 27487 (19.1) |  |
| **Maternal education** |  |  | P <0.001 |
| No education | 25052 (62.1) | 31747 (17.6) |  |
| Primary | 5686 (14.3) | 21052 (11.9) |  |
| Secondary | 8622 (19.9) | 90965 (52.4) |  |
| Higher | 1425 (3.7) | 27451 (18.2) |  |

P-value: Chi-square p-value. Weighted analysis using complex sample analysis.
